# How well have CMIP3, CMIP5 and CMIP6 future climate projections portrayed the recently observed warming

**DOI:** 10.1038/s41598-022-16264-6

**Published:** 2022-07-14

**Authors:** D. Carvalho, S. Rafael, A. Monteiro, V. Rodrigues, M. Lopes, A. Rocha

**Affiliations:** 1 CESAM-Department of Physics, University of Aveiro, Campus Universitário de Santiago, 3810-193 Aveiro, Portugal; 2 CESAM-Department of Environment and Planning, University of Aveiro, Campus Universitário de Santiago, 3810-193 Aveiro, Portugal

**Keywords:** Atmospheric science, Climate change

## Abstract

Despite the dire conclusions of the Intergovernmental Panel on Climate Change (IPCC) Assessment Reports in terms of global warming and its impacts on Earth’s climate, ecosystems and human society, a skepticism claiming that the projected global warming is alarmist or, at least, overestimated, still persists. Given the years passed since the future climate projections that served as basis for the IPCC 4th, 5th and 6th Assessment Reports were released, it is now possible to answer this fundamental question if the projected global warming has been over or underestimated. This study presents a comparison between CMIP3, CMIP5 and CMIP6 future temperature projections and observations. The results show that the global warming projected by all CMIPs and future climate scenarios here analyzed project a global warming slightly lower than the observed one. The observed warming is closer to the upper level of the projected ones, revealing that CMIPs future climate scenarios with higher GHG emissions appear to be the most realistic ones. These results show that CMIPs future warming projections have been slightly conservative up to 2020, which could suggest a similar cold bias in their warming projections up to the end of the current century. However, given the short future periods here analyzed, inferences about warming at longer timescales cannot be done with confidence, since the models internal variability can play a relevant role on timescales of 20 years and less.

## Introduction

Climate change impacts and adaptation for virtually all human activities are perhaps the greatest challenge mankind as ever faced. Regardless of the present and/or future measures taken to decrease greenhouse gases emissions, global climate change is currently well underway and its catastrophic effects have been impacting many countries in recent years. Climate change is responsible for most of the risk associated with weather related disasters worldwide^1^, and the increase in frequency and intensity of extreme events has been well documented in several studies (e.g.,^2,3^). Global warming has been speeding up in recent decades^4^, and extreme weather events such as heatwaves, droughts, floods, and wildfires are expected to increase in frequency, severity and intensity because of global warming^5–7^. A warmer climate with more droughts and higher winds will lead to an expansion of the fire-prone area and longer fire seasons^8^, and a potential decrease of the agricultural areas^9^.

The Intergovernmental Panel on Climate Change (IPCC) 6th Assessment Report^10^, the latest existent knowledge about the scientific, technical and socio-economic aspects of climate change, shows that it is virtually certain (> 95%) that human activity has been the main cause of the observed global warming since the mid-twentieth century. Other possible factors, such as natural internal variability of the climate system and natural external forcings (solar activity variations, volcanic activity, changes in Earth orbit, etc.), are considered to have a marginal contribution to global warming^11^. These human-induced climate changes are mainly forced by the continuously increasing emissions of GHG (mainly CO_2_) to the atmosphere. However, and although IPCC 4th^12^, 5th^13^ and 6th^10^ Assessment Reports showed conclusive evidences on human-induced global warming, there is still some skepticism and climate-change denial wave present in different communication and information platforms^14^, which spreads across a range of views, from complete denial of global warming to attempts to downplay the risks associated to climate change^15^. Although these are more commonly found among the less informed and/or specialized community, climate change skepticism has also been pervasive to academia, politics and decision-making, a cause of concern since it can act as a counterweight towards the adoption of effective measures to mitigate greenhouse gases (GHG) emissions. One of the claims of this skepticism is that simulated future climate projections are not able to realistically represent Earth’s climate response to increasing GHG emissions and, as such, the projected warming is alarmist, or at least exacerbated.

Future climate projections provide the fundamental basis for research about climate change, related impacts and mitigation measures. The IPCC Assessment Reports have been historically based on the Coupled Model Intercomparison Projects (CMIPs), a collaborative climate modelling framework coordinated by the World Climate Research Programme (WCRP). CMIPs provide present and future climate projections obtained from a wide range of Global Climate Models (GCMs), developed by the world-leading climate research institutes. CMIP’s Phase 3 (CMIP3^16^) and Phase 5 (CMIP5^17^) provided the present and future climatic data analyzed in IPCC’s 4th^12^ and 5th^13^ Assessment Reports, respectively. Currently, CMIP is in Phase 6 (CMIP6^18^), and its future climate scenarios served as basis for the recently released IPCC 6th Assessment Report^10^.

Considering that CMIP3, CMIP5 and CMIP6 future climate projections start in 2000, 2006 and 2015, respectively, it is presently possible to compare 21 years of CMIP3, 15 years of CMIP5 and 6 years of CMIP6 future climate projections with observations. This renders an unique opportunity to address the fundamental question if surface temperature changes projected by CMIPs is consistent with the ones observed in the last 2 decades. Although older future climate projections from IPCC 1st, 2nd and 3rd Assessment Reports were evaluated against observations in past studies (e.g.,^19–22^), more recent studies have compared newer CMIPs with observations but only regarding the past (historical) period and not the future projections (e.g.,^23–31^). Lewandowsky et al.^32^ analyzed a well-known alleged divergence between model projections and observations (the warming pause or hiatus) comparing CMIP5 RCP8.5 projected temperatures with observations up to 2016, and showed that there is no robust statistical evidence for the so-called divergence between projected temperatures and observations. However, this study only analyzed one future climate scenario (RCP8.5) from one CMIP (CMIP5). Thus, there is still lacking an objective and complete answer on how well future climate projections from the most recent CMIPs (CMIP3, 5 and 6) have portrayed recently observed surface temperature changes.

## Data and methods

Climate model simulations of surface temperatures are tipically compared with observation-based datasets of surface temperatures, but these are usually built considering measurements of air temperature over land and sea surface temperature (SST) over oceans. Such inconsistencies between observed and simulated datasets can account up to 25% of the differences between them^33^. Since many of the CMIPs GCMs used in the present study do not have available the SST variable (‘tos’), and bearing in mind that the human population lives exclusively over land areas, the present study will focus over land areas only.

Surface temperature data from a total of 15 CMIP3, 27 CMIP5 and 30 CMIP6 models, listed in Table 1, were analyzed in how they portray temperature changes over global land areas up to 2020.

**Table 1:** CMIPs GCMs.

CMIP3	CMIP5	CMIP6
NCAR-CCSM3	CMCC-CM	EC-Earth3
CSIRO MK3.0	MRI-CGCM3	EC-Earth3-CC
CSIRO MK3.5	CNRM-CM5	EC-Earth3-Veg
MPI-ECHAM5	MIROC5	AWI-CM-1-1-MR
GFDL-CM2.0	ACCESS1.0	MPI-ESM1-2-HR
GFDL-CM2.1	ACCESS1.3	CESM2-WACCM
BCCR-BCM2.0	HadGEM2-AO	CIESM
CNRM-CM3	HadGEM2-CC	CMCC-CM2-SR5
MIROC3.2	HadGEM2-ES	CMCC-ESM2
MRI-CGCM2.3	INM-CM4	NorESM2-MM
NCAR-PCM1	IPSL-CM5A-MR	TaiESM1
UKMO-HADCM3	CMCC-CMS	FGOALS-f3-L
IPSL-CM4	CSIRO-Mk3.6.0	BCC-CSM2-MR
CCCMA-CGCM3.1	MPI-ESM-LR	CAMS-CSM1-0
MIUB-ECHOG	MPI-ESM-MR	EC-Earth3-Veg-LR
GISS-MER	NorESM1-M	MRI-ESM2-0
INM-CM3.0	NorESM1-ME	CAS-ESM2-0
–	FGOALS-s2	MIROC6
–	GISS-E2-H	ACCESS-CM2
–	GISS-E2-H-CC	ACCESS-ESM1-5
–	GISS-E2-R	KACE-1-0-G
–	GISS-E2-R-CC	INM-CM4-8
–	IPSL-CM5A-LR	INM-CM5-0
–	IPSL-CM5B-LR	IPSL-CM6A-LR
–	–	KIOST-ESM
–	–	MPI-ESM1-2-LR
–	–	NESM3
–	–	IITM-ESM
–	–	FGOALS-g3
–	–	NorESM2-LM

For each CMIP, two climate scenarios were considered: a middle-of-the-road scenario with effective GHG emission reductions, and a business-as-usual scenario with virtually no GHG emissions reductions. These scenarios correspond to SRES B1 and SRES A2 for CMIP3^34^, RCP4.5 and RCP8.5 for CMIP5^17^, and SSP2-4.5 and SSP5-8.5 for CMIP6^18^, respectively. The projected (future) period, the one that is already a projection by the models, starts in 2000, 2005 and 2015, respectively for CMIP3, CMIP5 and CMIP6. For all CMIPs a 20-year period that precedes the start of the future projections was considered as historical, or baseline, period. For CMIP3, CMIP5 and CMIP6 the baseline periods are 1980–1999, 1986–2005 and 1995–2014, respectively.

CMIPs temperature data were compared with the Global Historical Climatology Network (GHCN) version 3 (described in^35^), a gridded data set of monthly mean global observations of surface temperatures, produced by the National Climatic Data Center (NCDC) of the National Oceanic and Atmospheric Administration (NOAA). Since GHCN only has available temperature data over land areas, all comparisons carried out in this study refer to land areas only. CMIPs and observed surface temperatures were compared up to 2020, in two different ways. First, in terms of time series of annual surface temperatures anomalies, A_year_, computed according to Eq. (1):

1 $${A}_{year}={\overline{T} }_{year}-{\overline{\overline{T}}}_{historical}$$

where ${\overline{T} }_{year}$ is the global mean temperature for a given year and ${\overline{\overline{T}}}_{historical}$ is the global mean surface temperature for the historical (baseline) period. Next, spatial maps of the differences between the observed and CMIPs-projected average warming, corresponding to each CMIP future period, were computed according to Eq. (2):

2 $$\Delta W={W}_{CMIP}-{W}_{OBS}$$

where ${W}_{CMIP}$ and ${W}_{OBS}$ are the annual surface temperatures anomalies averaged for the whole future period from CMIPs and observations, respectively, and calculated according to Eq. (3):

3 $$W = {\overline{A} }_{year}$$

For CMIP3, CMIP5 and CMIP6, $\Delta W$ was calculated over the period 2000–2020, 2006–2020 and 2015–2020, respectively.

## Results and discussion

**Figure 1:**
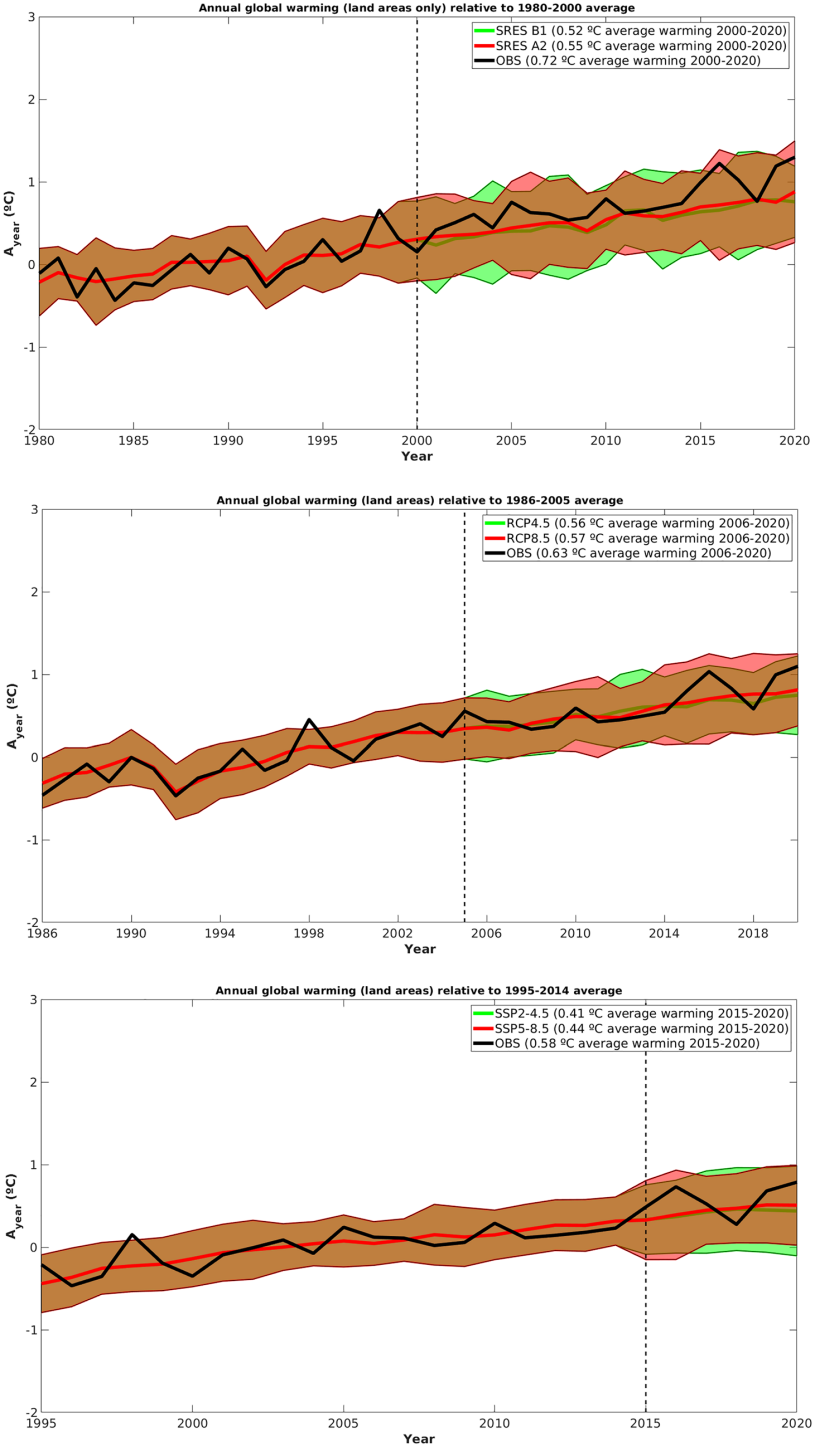
Annual global warming (land areas only) relative to the reference period: top panel for CMIP3, middle panel for CMIP5 and bottom panel for CMIP6.

Figure 1 shows time series of annual surface temperatures anomalies from observations and the CMIPs future climate scenarios. The solid colored lines represent the median anomalies among all models within each scenario, while the solid colored areas represent the median plus and minus two standard deviations of the anomalies among all models. The dashed vertical lines represent the start of the future climate projections for each CMIP.

Figure 1 shows that, for all CMIPs and scenarios, the observed warming generally fits well within the projected warming range, and closely follows the CMIPs median warming lines. The observed warming is usually closer to the upper level of the projected warming ranges. Considering the average warming for the whole future period, CMIP3 SRES B1 (SRES A2) average warming is 0.20 °C (0.17 °C) lower than the observed one, while for CMIP5 RCP4.5 and RCP8.5 the average warming is 0.07 °C and 0.06 °C lower than the observed one, respectively. CMIP6 SSP2-4.5 and SSP5-8.5 average warming is 0.17 °C (0.14 °C) lower than the observed one.

Thus, all CMIPs temperature projections up to 2020 are slightly conservative, particularly CMIP3. However, it should be noted that CMIP6 future period comprises only 6 years, which is relatively short to draw solid conclusions.

**Figure 2:**
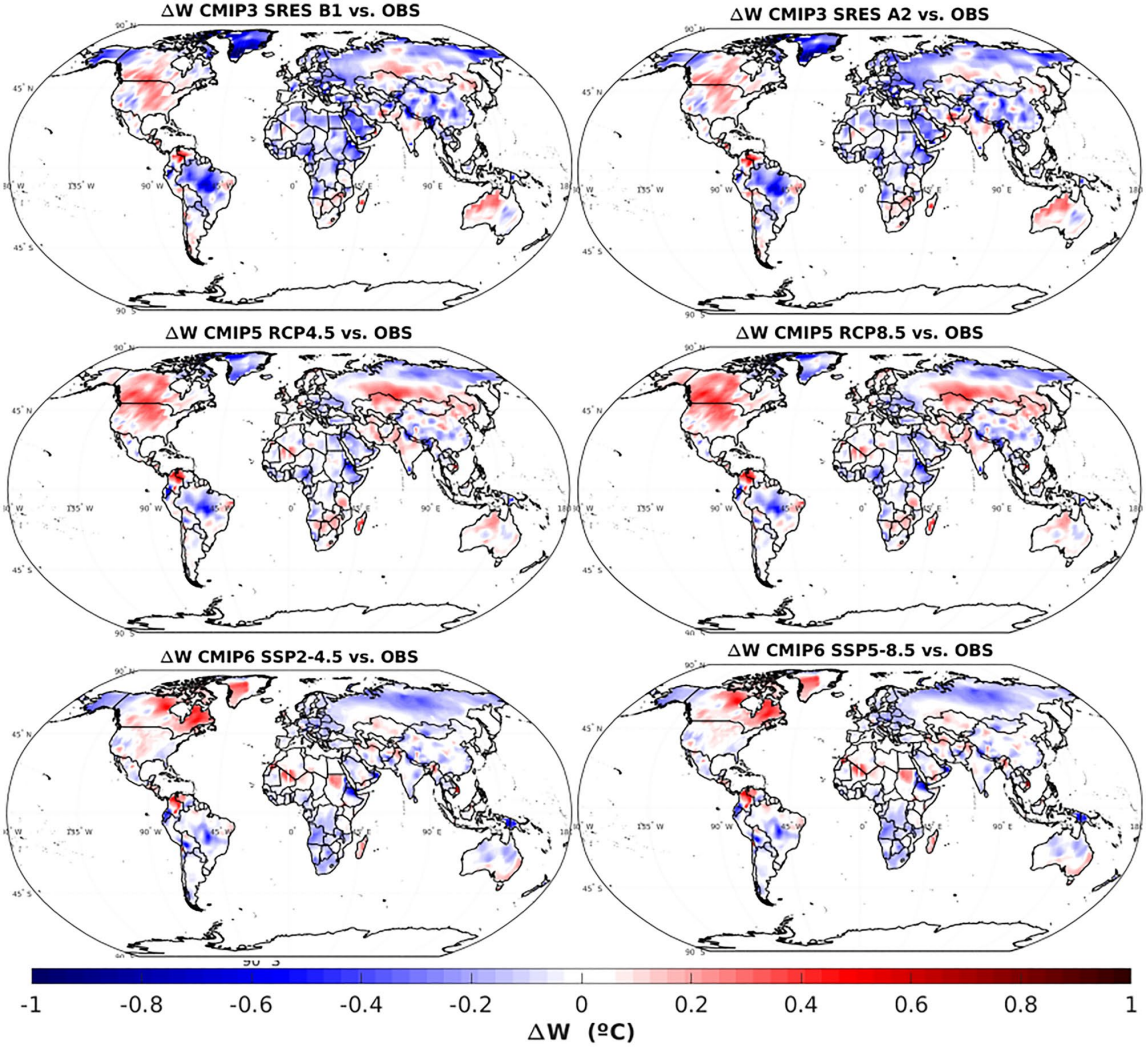
Spatial maps of the differences between the average warming projected by CMIPs and observations.

Figure 2 shows spatial maps of the differences between the average warming projected by CMIPs and observed, $\Delta W$.

Figure 2 shows that all CMIPs projected future surface temperatures are close to the observed ones, given that the differences between them are typically below 0.2 °C. Comparing the different CMIPs, it is clear that CMIP3 is the most conservative one, since it shows a substantial number of land areas where the projected warming was lower than what was observed, mainly at Greenland where the warming underestimation reached 1 °C. Other areas show an underestimation (Eurasia, central Brazil) or overestimation (central US–Canada, Colombia–Venezuela border and northern Australia) of the warming, but with small differences relative to the observations.

CMIP5 projected warming over land areas seems overall closer to observations than CMIP3, although some localized areas of the globe show relevant underestimations (eg., Greenland, central Brazil, northern Russia) or overestimations (US and Canada, Madagascar, Colombia–Venezuela and Russia–Kazakhstan borders). Looking at CMIP6, the differences between projections and observations are also low, except at Colombia-Venezuela border, Quebec and Nunavut areas in Canada (overestimation reaching 0.5 °C) and Russia and southern Africa (underestimation up to 0.5 °C). Within each CMIP, no major differences are seen between the two future temperature projections, which is expected since these differences tend to become more visible towards the end of the current century.

According to the results here presented, it is clear that the observations show a global warming tendency over land areas since 1980 to 2020. In terms of global means, all CMIPs and scenarios tend to slighly underestimate the observed warming. CMIP3 seems to be the one with the highest warming underestimation, while CMIP5 projected temperature increases are the ones more in line with the observations. In terms of the spatial variation of the warming, CMIP3 is again the most conservative one, showing a substantial number of land areas where the projected warming was lower than what was observed, mainly at Greenland. CMIP5 shows mixed results, with areas showing under and overestimation of the observed warming. However, the magnitude of these differences are relatively low.

## Conclusions

This study analyzed if future climate projections from the last three CMIPs realistically project global warming, by comparing 21 years of CMIP3, 15 years of CMIP5 and 6 years of CMIP6 future temperature projections with observations.

The results show that CMIPs future temperature projections here investigated portray a future temperature increase over land areas well in line, although slightly lower, than the observed one. CMIP3 seems to be the one with the highest warming underestimation, while CMIP5 is the one more in line with the observations. The observed warming is closer to the upper level of the projected ones, revealing that CMIPs future climate scenarios with higher GHG emissions appear to be the most realistic ones. Spatially, no major differences are seen between CMIPs projections and observations: some land areas show positive or negative biases, but they are relatively low except over Greenland in CMIP3 (strongest warming underestimation) and US-Canada in CMIP5 (largest overestimation).

These results show that CMIPs future warming projections have been slightly conservative up to 2020, which could suggest a similar cold bias in their warming projections up to the end of the current century. However, given the short future periods here analyzed, inferences about warming at longer timescales cannot be done with confidence, since the models internal variability can play a relevant role on timescales of 20 years and less.

Finally, it should be noted that only the future temperature projections from CMIPs scenarios are under evaluation in this study, not the scenarios per se.

## Data Availability

All CMIP6 datasets analysed in the current study are publicly available in the Earth System Grid Federation (ESGF) Data Portal: https://esgf-node.llnl.gov/projects/cmip6/. The Global Historical Climatology Network (GHCN) version 3 data is also publicly available at: https://www.ncei.noaa.gov/products/land-based-station/global-historical-climatology-network-monthly.
